# Influence of the asthenosphere on earth dynamics and evolution

**DOI:** 10.1038/s41598-023-39973-y

**Published:** 2023-08-17

**Authors:** Lawrence Cathles, Willy Fjeldskar, Adrian Lenardic, Barbara Romanowicz, Johnny Seales, Mark Richards

**Affiliations:** 1 Department of Earth and Atmospheric Sciences, Cornell University, Ithaca, USA; 2 Tectonor, Stavanger, Norway; 3 Department of Earth Science, Rice University, Houston, USA; 4 Department of Earth and Planetary Science, University of California, Berkeley, USA; 5 Department of Earth and Space Sciences, University of Washington, Seattle, USA

**Keywords:** Solid Earth sciences, Geodynamics

## Abstract

The existence of a thin, weak asthenospheric layer beneath Earth’s lithospheric plates is consistent with existing geological and geophysical constraints, including Pleistocene glacio-isostatic adjustment, modeling of gravity anomalies, studies of seismic anisotropy, and post-seismic rebound. Mantle convection models suggest that a pronounced weak zone beneath the upper thermal boundary layer (lithosphere) may be essential to the plate tectonic style of convection found on Earth. The asthenosphere is likely related to partial melting and the presence of water in the sub-lithospheric mantle, further implying that the long-term evolution of the Earth may be controlled by thermal regulation and volatile recycling that maintain a geotherm that approaches the wet mantle solidus at asthenospheric depths.

## Introduction

The asthenosphere concept dates from early studies of isostasy^1^, bolstered by the existence of a seismic “low velocity zone or LVZ”^2^ beneath what we now understand to be the lithosphere. The upper boundary of the asthenosphere is commonly referred to as the “Lithosphere Asthenosphere Boundary” (LAB) as defined by numerous seismological studies. However, the asthenosphere is often considered to extend to greater depths than the LVZ, since its lower boundary is in general not well-constrained by geophysical observations In the sections that follow we mostly consider the asthenosphere to be a thin and very weak sub-lithospheric layer, as distinguished from the general notion that the upper mantle is on average less viscous than the lower mantle, and as indicated by numerous studies following the advent of global seismic tomography^3–10^. In general, the fine radial structure of upper mantle viscosity is not resolved in such global studies, but *Richards and *Lenardic^11^ have recently shown that a thin, more fluid asthenosphere can accommodate most geophysical constraints as well as can a less fluid upper mantle, and that a thin asthenosphere is much more effective in facilitating plate tectonics.

The purpose of this paper is to elaborate upon the broad applicability of this perspective, independent of the question of the “average” viscosity contrast between the upper and lower mantle. We summarize evidence from several geophysical disciplines that indicates that the asthenosphere is ubiquitous; thin (< 300 km thick); not equivalent in function to (e.g., cannot be replaced functionally by) a thick lithosphere; essential to plate tectonics and to understanding how plate tectonics was established and is maintained; and provides insights into volatile recycling in the mantle. Particularly in the glacial isostatic adjustment section immediately below, old data are revisited with simple models to highlight key issues we believe have been overlooked or forgotten. Evidence from other disciplines is then examined to argue for the existence and broad importance of a thin asthenosphere. Our intention is not to review past work, but rather, on the basis of past and ongoing work, to present a new and broad perspective on the asthenosphere and upper mantle that we believe is important to geodynamic studies and worthy of consideration as, effectively, a “starting model” for treating a broad range of geophysical problems.

## Glacio-isostatic adjustment (GIA) and other transient loading problems

### Glacial isostatic adjustment

Evidence for an asthenosphere comes from the straightforward observation that the isostatic adjustment of small-scale loads is far more rapid than might be inferred from the response to large-scale loads. Since small-scale loads induce flow only to shallow depths, one explanation is that the earth’s shallow mantle is more fluid. Another is that stresses arising from the flexure of the lithosphere accelerate the response to small-scale loads more than to large-scale loads. The relative role of the lithosphere and asthenosphere in accelerating isostatic adjustment is first addressed here using long established and accepted emergence data from Fennoscandia and simple models plotted in a fashion that separates data and interpretation. GIA evidence for the widespread presence of an asthenosphere is then briefly reviewed. We note that the Fennoscandian data are most appropriate in these regards precisely because they continuously span the transition from long-wavelength to short-wavelength behavior that best illuminates the influence of a thin, weak asthenosphere.

**Figure 1:**
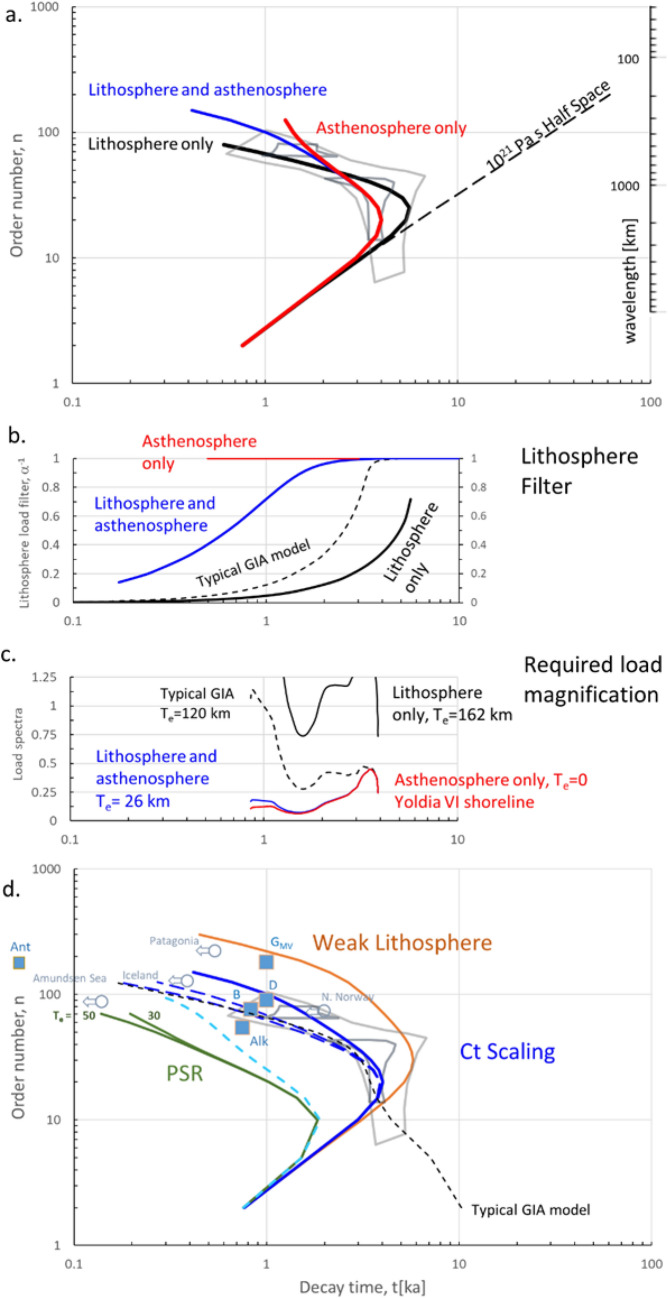
The earth’s isostatic response to loading as characterized by the decay time of harmonic load components. Grey polygons or data points show observational data. Curved lines show the calculated response to loading of earth models defined in Table 1. Curves with the same color are for the same earth model. The abscissa (decay time) axis is the same for all panels. (**a**) The large grey polygon outlines the relationship between decay time and uplift wavelength (right axis) or order number (left axis) determined by McConnell’s ^12^ Hankel transform of the uplifted shorelines in Fennoscandia mapped by Sauramo^14^. Enclosd smaller grey polygons show Mitrovica and Peltier’s^15^ more recent analysis of the same data. Red, black and blue lines plot the calculated loading decay times as a function of load wavelength for earth models defined in Table 1. (**b**) Fraction of the surface load compensated by vertical deflection of the lithosphere. (**c**) The surface load magnification needed to produce the shoreline uplift spectrum of Sauramo and McConnell (red curve). The curves in this panel are the result of dividing the red curve in this panel by the lithosphere filter curves in (**b**). The red curve shows the amplitudes of the Hankel transform of the 9200 ybp YV1 shoreline from McConnell (1968, Fig. 4) plotted against decay time. (**d**) Same as (**a**) but showing additional load response curves and observations. Boxes plot decay times inferred from uplifted shorelines, circles plot the maximum decay times inferred from GPS uplift rates in areas de-glaciated in the last few hundred years. Discussion and references given in SM2 and SM3. Ant = South Shetland Islands in north West Antarctica, Alk = SE Alaska, D = Devon Island Antarctic Canada, B = Lake Bonneville Utah, G_MV_ = Greenland Meisters Vig area.

Figure 1 shows the log_10_ amplitude decay time (abscissa) of sinusoidal loads of different wavelength (order number) imposed on the earth’s surface. Load wavelength decreases upward, and response time increases to the right. The figure (due originally to McConnell^12^ and used in Cathles^13^) is powerful because observations (shown as data points and grey polygons) are completely independent of the models that potentially explain them. Model predictions are indicated by solid and dashed curves; data by grey polygons and data points.

The largest *grey polygon* in Fig. 1a encloses the response times inferred by McConnell^12^ by Hankel transforming (Fourier transforming in cylindrical coordinates) uplifted shorelines mapped by Sauramo^14^ in Fennoscandia (Finland, Sweden, Norway and Denmark). Paleo-shoreline elevations (such as the Yoldia Sea VI shoreline we will discuss below) were mapped across Fennoscandia in the early 1900’s and the shoreline ages determined by varve count. These data remain the best for defining the pattern of regional uplift and its history. Sauramo noted that the response to unloading in the central Gulf of Bothnia region was initially more rapid than the broader response to the deglaciation. In a more recent analysis, Mitrovica and Peltier^15^ emphasized this bimodal character of the Fennoscandian isostatic response with two smaller polygons (the grey polygons enclosed by McConnell’s larger polygon). The upper left polygon represents a small peak in the decay spectrum with a decay time of ~ 1 ka and a load wavelength of ~ 500 km. The lower polygon indicates a decay time of 4.5 ka and load wavelengths up to ~ 4000 km. The grey polygons hook dramatically upward to the left, showing clearly that the shorter wavelength load components decay much more rapidly than the longer wavelength components.

The curves shown in Fig. 1a depict the response to harmonic loading calculated (see Supplementary S1) for a 3-layer earth (lithosphere, asthenosphere, and half-space mantle) with parameters listed in Table 1.

**Table 1:** Parameters describing a three-layer earth model used to compute the curves in Fig. 1 (see Supplementary S1). F_r_ is the flexural rigidity of the lithosphere, T_e_ is the corresponding effective elastic thickness of the lithosphere, H_asth_ is the thickness of the asthenosphere, η_asth_ its viscosity, and η_m_ is the viscosity of the mantle underlying the lithosphere.

Figure	Model	F_r_ (10^23^ Nm)	T_e_ (km)	H_asth_ (km)	η_asth_ (10^21^Pa s)	η_m_ (10^21^Pa s)
1a, b, c	Asthenosphere only (red)	0	0	75	1.5 × 10^–2^	1
	Lithosphere only (black)	500	162	0	–	1
	Fennoscandia (blue)	2	26	75	1.5 × 10^–2^	1
1d	Fennoscandia (dash turq.)	2	26	150	1.5 × 10^–2^	1
	PSR (upper green)	13	50	80	2 × 10^–3^	1
	PSR (lower green)	2.9	30	80	2 × 10^–3^	1
	Ct scaling (blue) as above	2	26	75	1.5 × 10^–2^	1
	½ H_asth_	”	”	37.5	1.9 × 10^–3^	”
	¼ H_asth_	”	”	18.75	2.3 × 10^–4^	”
	Weak lithosphere (brown)	0.5	16	74	4 × 10^–2^	1
	Typical GIA (dashed black)	201	120	650	0.4	20

The earth is spherical, and for order numbers (conceptually the number of wavelengths (λ) encircling the globe) smaller than ~ 11, motions of the core-mantle boundary and changes in gravity are important. Since we are concerned here with the asthenosphere and upper mantle, this low order number response is not important, and we can best illustrate the points we wish to make with an analytic 3-layer Cartesian earth model.

The three-layer model consists of a lithosphere characterized by its flexural rigidity (or equivalently its effective elastic thickness), an asthenosphere with specified thickness and viscosity, and a half-space mantle below the asthenosphere with specified viscosity. The asthenosphere and mantle are purely viscous. The relaxation time of an imposed harmonic load is *h*(*t*) =*h*_*o*_ *α*^−1^* exp*(− *t*/(*τ*_*m*_* α*^−1^* R*), where the load beneath the lithosphere is the surface load reduced by *α*^−1^, and the exponential decay time *τ*_*m*_ is reduced by *α*^−1^* R*. *τ*_*m*_ is the relaxation time of the mantle (e.g., an earth with no lithosphere or asthenosphere), *α*^−1^ is the fraction of the surface load that is not supported by the lithosphere (the range from full to no support is 0–1), and *R* is the reduction in *τ*_m_ by asthenosphere flow. *α*^−1^ is a function of the load wavelength and the flexural rigidity, and can be thought of as a load filter. The flexure of the lithosphere accelerates the isostatic response because elastic as well as gravity stresses drive flow in the asthenosphere and mantle. The actual thickness of the lithosphere is not specified by this GIA model. It might be the 77 km thickness of the crust and lid in PREM^16^, or it might be larger. Lithosphere thickness is not well know at present, even though observations constrain the flexural rigidity of the lithosphere.

Comparison of the calculated curves and grey outlined data in Fig. 1a shows two important things: First, the observed Fennoscandian relaxation spectrum is matched equally well by an earth that has only a very thick lithosphere (black curve), an earth with only an asthenosphere (red curve), or an earth with both a lithosphere and an asthenosphere (blue curve). All three of these curves pass though the grey data polygons. This demonstrates that a very wide range of earth models can produce decay spectra compatible with the observations (the grey polygons). Second, the changes in decay time with load wavelength are significant. The response of shorter wavelength loads in Fennoscandia is dramatically faster than expected for a uniform 10^21^ Pa s half space (dashed line). At n = 100 (*λ* = 398 km), for example, the decay time with no lithosphere or asthenosphere would be ~ 30 ka, but the response time indicated by McConnell’s data is ~ 1 ka. For the 4 × 10^20^ Pa s upper mantle viscosity favored in many GIA analyses, the response time would be 12 ka, but this is still much greater than 1 ka. The rate of isostatic adjustment is dramatically accelerated by the lithosphere/asthenosphere combination.

Figure 1b shows that, although the curves in Fig. 1a all have a spectral response compatible with McConnell’s constraints on relaxation time vs. wavelength, the surface loads required to produce them are *very* different. Reasonable surface loads of short wavelength induce very little vertical displacement of a thick lithosphere. For example, the black “lithosphere only” curve in this panel shows that only ~ 5% of an applied surface load is compensated by downward deflection of the lithosphere at the wavelengths of the fast-response spectral peak at *τ* ~ 1 ka. To produce the response observed in Sauramo’s data, its short wavelength surface load components would have to be increased an implausible 20 fold in a lithosphere-only model.

Figure 1c emphasizes and extends this observation. The curves shown are the Yoldia VI shoreline spectrum curve divided by the lithosphere load filter *α*^−1^ from Fig. 1b. If there is no lithosphere, *α*^−1^ = 1 at all load wavelengths and decay times, and the full surface load drives flow in the asthenosphere and mantle. The downward deflection of the lithosphere caused by the load is reflected in the uplift of the Yoldia VI shorelines when the load is removed (the red curve). If there is a thin lithosphere, the surface load must be augmented only slightly at short wavelengths, as shown by the blue curve (produced by division of the red spectra curve by the blue “lithosphere and asthenosphere” curve in Fig. 1b). On the other hand, the lithosphere of a typical GIA earth model with an effective elastic thickness of 120 km would require a completely unreasonable surface load amplification at shorter decay times and wavelengths (the dashed black curve) to produce the observed Yoldia VI shoreline uplift. The small peak at *τ* = 1 ka, for example, represents a rapid early uplift of ~ 40 m which could be attributed to ~ 120 m of water unloading if the lithosphere is thin (blue curve), but a thick lithosphere (black dashed curve) would require 800 m of water unloading, which is not geologically plausible. For elaboration see Sect. S2.

Because the lithosphere accelerates the isostatic response, the “typical GIA earth model” (2 × 10^22^ Pa s lower mantle, 0.4 × 10^21^ Pa s upper mantle, no asthenosphere, and high flexural rigidity lithosphere) passes through the grey data polygons (dashed black curve in Fig. 1d). The blue “lithosphere and asthenosphere” does also, but it is calculated for a very different earth model (10^21^ Pa s mantle, a thin fluid asthenosphere, and a low flexural rigidity lithosphere). In fact, all 23 of the quite different Fennoscandian earth models by different workers tabulated by Steffen and Wu^7^ pass through or close to the grey data polygons, filling the space between these two cases, as shown by Fig. S3.1. The spectra of these models differ mainly at low order numbers. Modelers are free to choose very different lower mantle viscosities because the Fennoscandian glacial system is not large enough for the isostatic response to its melting to be affected by the viscosity of the lower mantle. Lower mantle viscosity is only probed by very long wavelength load redistributions such as the transfer of the glacial ice load to the oceans when they melt. The viscosity of the lower mantle is an open question and could be as low as ~ 10^22^ Pa s^4^. In any case, upper mantle earth models with a wide range of lithosphere, asthenosphere, and mantle (to ~ 1000 km depth) properties are spectrally equivalent.

The important difference between the earth models is the effective flexural thickness of their lithospheres. To some extent, the load supported by a lithosphere can be compensated by increasing the ice thickness. It is customary to tailor the ice load history used with a particular earth model so that isostatic predictions better match observations, and these ice thickness modifications can be substantial. For example, the thickness of Fennoscandian ice loads at the Last Glacial Maximum differ by more than a factor of 2, depending on the earth model used (Steffen and Wu^7^, Fig. 14).

The essential point that we wish to make here is that a thick lithosphere cannot substitute for an asthenosphere, because reasonable short wavelength surface loads would drive insufficient mantle flow, and the increases in ice load required to cure this situation are too large. If a fast isostatic response is observed at short wavelengths, an asthenosphere is indicated. Although the data we are using here are not new, this critical point appears not to have not been sufficiently appreciated in the intervening time. Lithosphere load support and banded gravity anomalies with wavelength of 200–400 km were the initial basis for thinking that an asthenosphere is present under Fennoscandia^13^, but the presence of an asthenosphere is supported by the rapid isostatic adjustment of the northern Gulf of Bothnia area discussed above. Recent high-resolution modeling has shown that the tilt of shorelines along the Norwegian coast^17^ and the isostatic response to Younger Dryas glacial advances near Bergen^18^ require a thin upper mantle asthenosphere and a lithosphere with low flexural rigidity (T_e_ = 26 km). We believe that this evidence is compelling, and, unfortunately, often overlooked.

The other curves and data points in Fig. 1d show that observations at other locations also indicate the presence of an asthenosphere. *Blue squares* indicate the isostatic decay times inferred from shorelines uplifted in response to the removal of glacial loads that are small (< 150 km) in at least one dimension. The small-scale unloading data points plotted all have decay times less than 1 ka. For example, the melting of the last ice of the last glaciation on Devon Island produced an emergence of ~ 85 m with a decay time of ~ 1 ka. The melting of Little Ice Age glaciers in the South Shetland Islands in northern West Antarctica produced ~ 7 m of emergence with a response time of ~ 70 years^19^. The decay times are plotted at the order number that characterizes the response at the center of the unloaded area (see Supplementary S1). References and additional information are provided in a table in Supplementary S4.

Rapid GPS uplift rates of 2–40 mm/year are observed in almost all locations of recent deglaciation, most notably where Little Ice Age (LIA) glaciers have melted or are melting. The maximum response times indicated by these observations are plotted as *open circles* in Fig. 1d. The observed uplift rates are much greater than can be explained by an elastic response to unloading, require an asthenosphere, and often require a lithosphere considerably weaker than the 1D seismic earth model PREM^16^. The flexural rigidity computed from the PREM model, which has a 77 km thick lithosphere, is 53 × 10^23^ Nm. The flexural rigidities inferred from GIA (see Supplementary S1) are often much less than 53 × 10^23^ Nm, and consequently they suggest elastic lithosphere thickness much less that 77 km. These are referred to as “effective” flexural thicknesses, *T*_*e*_. For example, an uplift rate of 23 mm/year is observed near the largest glacier in Iceland (the Vatnajokull ice cap) which lost ~ 40 m of ice since the end of the LIA at ~ 1890 AD, and the effective lithosphere thickness indicated by modeling is *T*_*e*_ = 13–35 km^20^. The lithosphere thicknesses in parts of Greenland and Patagonia are also small, perhaps indicating an effective flexural thickness as low as 16 km (weak lithosphere curve). The uplift rate in the Amundsen Sea Embayment in Antarctica is 41 mm/year (4 × faster than the uplift in central Fennoscandia!) and requires an asthenosphere with a viscosity < 1.6 × 10^19^ Pa s and *T*_*e*_ = 60 km. Fast isostatic response times are of special interest in Antarctica because rapid uplift can keep glaciers grounded and prevent ice sheet collapse under moderate warming scenarios^21^. Additional references and discussion are provided in Supplementary S5.

Figure 1d highlights two other important observations. First, the dashed blue curves show the changes that result if the asthenosphere thickness of the solid blue curve is decreased by 2 and 2^2^ while its viscosity is decreased by 2^3^ and 2^6^ (Table 1). The response curves are nearly the same, as predicted by the *Ct* scaling developed by Richards and Lenardic^11^, in which decay time scales inversely with the cube of the thickness. Differences arise (i.e., the curves do not perfectly overlap) because the criterion for this *Ct* scaling, that the asthenosphere be thin compared to the load wavelength, is not fully met. The point again is that a broad range of Glacial Isostatic Adjustment (GIA) Earth models can be effectively equivalent in terms of their relaxation responses.

Second, the green curves in Fig. 1d are calculated from parameters inferred from post-seismic rebound (PSR) in Indonesia determined by Hu et al.^22^, and the turquois curve is the Fennoscandia Earth model with a thick asthenosphere. These curves show that post-seismic rebound indicates a response spectrum not too different from that of glacial isostatic rebound.

In summary, this section shows that earth models with a very wide range of properties can all match the spectral isostatic response observed in Fennoscandia. However, earth models with thick lithospheres cannot produce the observed short wavelength isostatic response because too much of the surface load is supported by the lithosphere. A thin asthenosphere is required by rapid responses to loading observed in cratonic Fennoscandia and Canada, and in almost all locations recently deglaciated. The global ubiquity of an asthenosphere is suggested by other data, such as PSR and especially seismic anisotropy, to whose discussion we now turn.

### Post-seismic rebound evidence for an asthenosphere

Post-seismic rebound (PSR) is to first-order determined by viscous creep in the sublithospheric mantle following earthquakes large enough to generate geodetically-measurable long-term deformation. The scaling of PSR in terms of asthenospheric properties differs from the scaling for GIA, geoid, and mantle convection studies^11^. This difference, fortuitously, allows for PSR studies to be combined with these other observational approaches to help independently determine asthenospheric thickness and viscosity contrast, in particular, their ability to resolve shallow mantle absolute viscosity. Although sophisticated models include the complicated effects of an elastic lithosphere and viscosity layering, the timescale for PSR basically scales as asthenosphere viscosity divided by asthenosphere thickness (η/*h*)^23^. PSR studies reveal absolute asthenosphere viscosities of order 10^17^–10^19^ Pa s^22,24,25^, in some cases significantly lower than inferred from the short-wavelength GIA studies discussed above. This broad range of viscosities is not considered controversial among PSR researchers, and there is significant variation depending upon differing tectonic environments. Some PSR studies have been interpreted to suggest that transient or non-linear (power-law) rheology may be at play, so that there is a time-dependence of rheology, with shorter timescales of post-seismic deformation suggesting smaller effective viscosity immediately beneath seismically-active lithosphere^26,27^. This may in turn explain why some PSR studies imply uppermost asthenospheric viscosities even lower than derived from short-wavelength GIA studies. Because PSR studies are generally sensitive to the viscosity of the uppermost sublithospheric mantle (perhaps only ~ 10 km or so), these low values are not inconsistent in any obvious way with, e.g., the Fennoscandian rebound results discussed above, as per the restrictions implied by the *Ct scaling*. Also, Boulze et al.^28^ have recently concluded that the asthenosphere responds to large subduction zone earthquakes via linear (Newtonian) rheology, as opposed to power-law rheology.

Figure 1d (green curves) and Table 1 (curves labeled as green) refer to sub-lithospheric mantle viscosity structure determined from the study of post-seismic relaxation following the M_w_ 8.6 Indian Ocean earthquake of 2012^22^. The GIA curves in Fig. 1 are calculated from this structure, and indicate even smaller short-wavelength relaxation times than inferred, e.g., from Fennoscandian GIA. This inference is, however, consistent with GIA models for very thin (~ 100 km) and low viscosity (~ 10^18^ Pa s) asthenosphere, as per the *Ct* scaling of Richards and Lenardic^11^. We note that other short-wavelength studies, perhaps most famously the rebound of Pleistocene Lake Bonneville^29^ are also consistent with such inferences. Therefore, suffice it to say that PSR studies are consistent with a very thin and weak asthenosphere, and furthermore should, in principle, enable finer resolution of the thickness vs. viscosity contrast tradeoff across all geodynamic studies.

### A brief note on geoid modeling constraints

Since the advent of global seismic tomography many studies have sought to use joint modeling of inferred 3-D mantle density structure and the Earth’s long-wavelength gravity field, or geoid, to constrain the radial viscosity structure of the mantle^30–32^. These studies suggest an upper mantle that is on average one to two orders of magnitude lower viscosity than the lower mantle. However, long-wavelength geoid modeling is inherently incapable of resolving fine-scale radial viscosity structure, and shorter-wavelength studies are compromised by lateral viscosity variations in terms of resolution of upper-most mantle viscosity structure^33,34^, and, like long-wavelength GIA studies^35^, they are very limited in terms of ability to resolve fine-scale radial viscosity structure^36,37^. Shorter-wavelength geoid studies are also strongly limited by the complicated rheological effects of plate boundaries, where short-wavelength geoid anomalies are most prominent^36^. A more detailed discussion of this challenge is given by Richards and Lenardic^11^ as a type example of the restrictions implied by the Cathles parameter (*Ct*) limitation on independently resolving asthenosphere thickness and viscosity contrast, so for the present purposes suffice it so say that geoid modeling studies are *consistent* with a very thin and very weak asthenosphere, but do not offer more precise constraints than GIA studies (previous section) and mapping of seismic anisotropy (next section).

### Seismic anisotropy beneath oceanic and continental plates

The major component mineral of the upper mantle, olivine, is strongly elastically anisotropic^38,39^ and likely deforms in the dislocation creep regime down to depths of 200–400 km^40^, resulting in seismic anisotropy at the macroscopic scale. Over long-time scales, olivine crystals tend to align their fast [100] axis predominantly in the direction of the prevailing mantle flow, as predicted from laboratory studies^41,42^ and numerical modeling^43,44^, and as observed in ophiolites^45^. At the macroscopic scale, seismic observations of anisotropy are often simplified into two types: (1) radial, or polarization anisotropy (also called “transverse isotropy”), with a vertical symmetry axis, measured from the discrepancy between the velocities of surface waves polarized horizontally and vertically, and (2) azimuthal anisotropy, with a horizontal axis of symmetry, measured from body wave splitting data and the azimuthal dependence of surface wave velocities. Radial anisotropy (resp. azimuthal anisotropy) is obtained from the azimuth independent (resp. dependent) part of seismic wave travel time measurements. It is now widely accepted that observations of shear wave splitting and surface wave azimuthal and radial anisotropy can be used to infer shear deformation and the direction of mantle flow at global and plate scales, either in the past from anisotropy "frozen" in the lithosphere, or in the present from anisotropy currently in the asthenosphere^46^.

While the simple relationship between direction of flow and seismic anisotropy may be altered in the presence of water^47,48^ or other upper mantle minerals^49^, the correlation of fast axis of anisotropy with the direction of past or present convective mantle flow has been documented repeatedly in the oceanic upper mantle, starting with the pioneering work of Hess^50^ and Morris et al.^51^ on the azimuthal variations of P_n_ velocities, and Forsyth’s^52^ documentation of the consistent azimuth of anisotropy in long period Rayleigh waves crossing the Pacific Basin.

**Figure 2:**
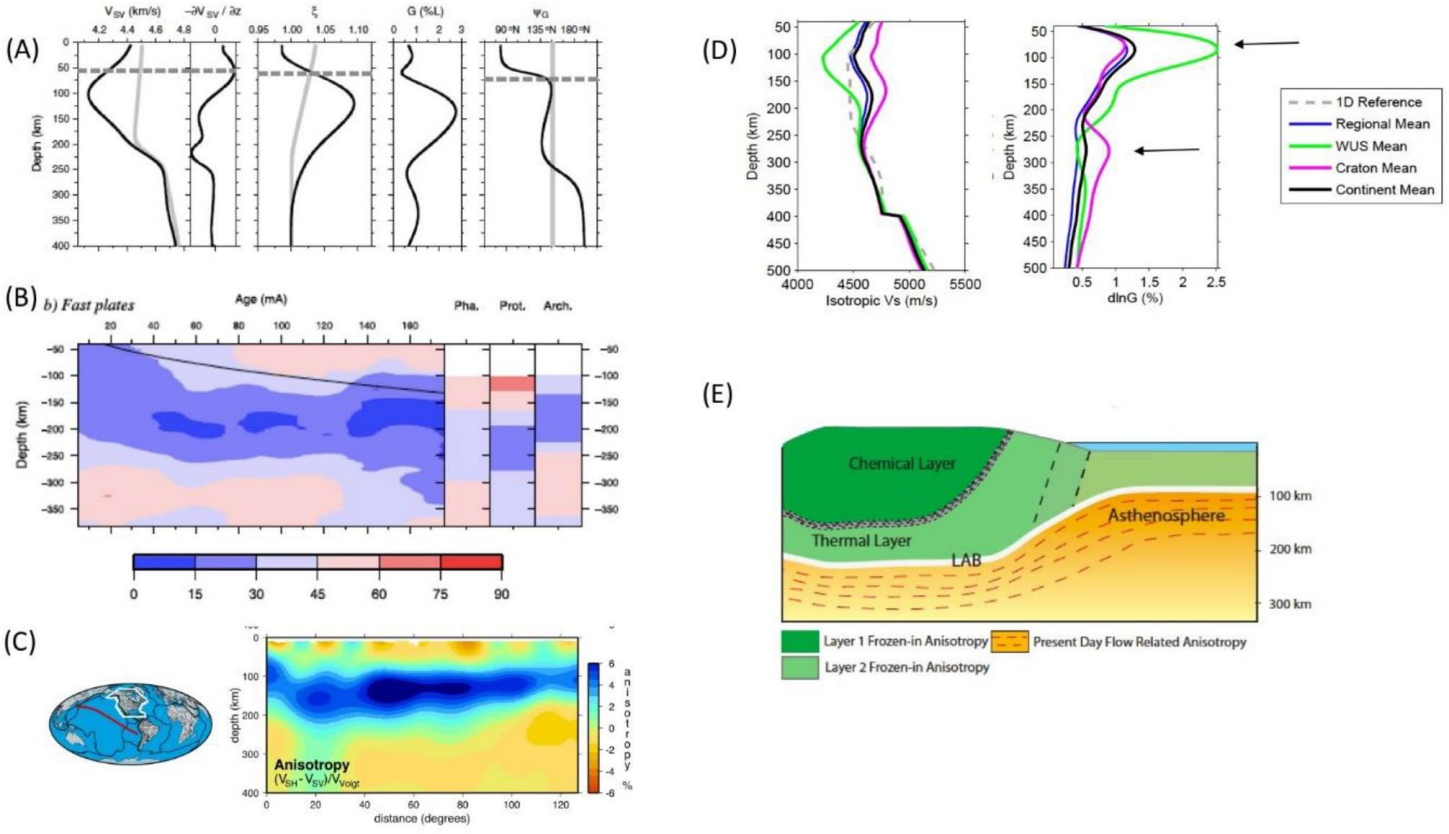
Evidence for distinct seismic anisotropy signature in the lithosphere and asthenosphere. (**A**) Depth profiles of, from left to right: Vsv, gradient of Vsv, radial anisotropy parameter ξ, strength of azimuthal anisotropy G, respectively (grey lines indicate starting model in study) and direction of fast axis of anisotropy Ψ_G_ at a point in the middle of the Pacific ocean (lat 35.03° W, lon 35° N).In the latter case the grey line indicates the direction of APM of the Pacific Plate in the (NNR)NUVEL-1 model. Horizontal broken line indicates the approximate depth of the LAB Adapted from Burgos et al.^57^; (**B**) Depth dependence of the average angular difference between the fast axis of azimuthal anisotropy and the APM beneath fast moving plates; left: for oceanic plates as a function of age; right: for continental plates divided into Phanerozoic (Pha), Proterozoic (Prot) and Archean (Arch.). From Debayle and Ricard^55^; (**C**) Depth cross section in the Pacific Ocean showing channel of strong radial anisotropy with Vsh > Vsv in the depth range ~ 100–250 km across the entire width of the ocean^63^. LAB; (**D**) Average isotropic velocity depth profiles (left) and strength of azimuthal anisotropy parameter G (right) for the north American continent^77,79^, comparing the mean over the western US (WUS, green) where the lithosphere is thin and the cratonic region (pink) where the lithosphere is ~ 200–250 km thick, to the regional and continental average. Arrows indicate the maxima in the strength of anisotropy. (**E**) Cartoon from Yuan and Romanowicz^77^.

Studies of surface wave azimuthal anisotropy in ocean basins consistently find fast directions quasi-parallel to the fossil spreading direction in the lithosphere and quasi-parallel to the present-day absolute plate motion (APM) at asthenospheric depths^53–56^. A rapid change in direction of the fast axis of seismic anisotropy as a function of depth, towards a direction quasi-parallel to the absolute plate motion, as inferred from Rayleigh waves (Fig. 2: panel A), has been interpreted as marking the lithosphere-asthenosphere boundary (LAB)^56,57^. Global studies that average structure at ocean basin scales generally find stronger azimuthal anisotropy in the asthenosphere than in the lithosphere, with a maximum around 100–150 km depth in the middle of the upper mantle seismic low velocity zone (LVZ), tapering off at depths greater than 250–300 km (Fig. 2: panel B). This coincidence of strong anisotropy with the depth range of the LVZ is one of the reasons why the LVZ is usually associated with the asthenosphere, at least in the minds of seismologists. Recently, departures from this simple model have been documented at the local scale in the middle of the Pacific plate^58,59^ with maxima in azimuthal anisotropy reported to occur at the top and the bottom of the LVZ, perhaps suggesting Poiseuille flow in the asthenosphere (as discussed in the next section).

These relationships are not restricted to oceanic areas. The difference in propagation velocity of surface waves polarized vertically (V_sv_, Rayleigh waves) and those polarized horizontally (V_sh_, Love waves) indicates radial anisotropy. This anisotropy is commonly captured in the parameter ξ = (V_sh_/V_sv_)^2^. Horizontal alignment of the fast axis of olivine, suggesting horizontally oriented flow, is reflected in observations of ξ > 1, while regions with ξ < 1 are interpreted as indicating the presence of quasi-vertical flow^60,61^. While radial anisotropy with ξ > 1 can reflect fine scale layering below the resolution of long wavelength surface waves^62^, the fact that ξ > 1 is so generally observed in the first ~ 200–250 km of the mantle at the global scale^16^ is widely attributed to indicate a prevailing horizontal flow in the top boundary layer for mantle convection^63^, with a channel of strong ξ > 1 roughly coinciding with the upper mantle LVZ. The bottom of this channel is generally found at a relatively constant depth of 250–300 km, while lateral variations in the depth of its top limit are attributed to the variations in thickness of the lithosphere^64,65^.

Under continents seismic anisotropy can be obtained not only from surface wave measurements, but also from shear wave splitting observations^66^. Two layers of radial or azimuthal anisotropy are often reported, one in the depth range corresponding to the lithosphere, and the other in the asthenosphere, with strong azimuthal anisotropy aligned with the APM at the base of the plate. This is observed in Australia^67,68^, and in the Baltic Shield^69^. Radial anisotropy with ξ > 1 is also found in these areas at asthenospheric depths, with a possible peak in amplitude at around 200 km depth^70,71^.

Fundamental mode surface waves lose resolution below 200 km depth, which requires the use of overtone measurements to resolve anisotropy in the asthenosphere below continents, while SKS splitting measurements provide denser spatial resolution, but have poor resolution in depth. In the north American continent, a long debate arose on whether the observed SKS splitting should be attributed to frozen in anisotropy^72^ or present day asthenospheric deformation^73^ or a combination of both^74,75^. A recent study of SKS splitting based on the recordings of the dense USArray points to a significant component of azimuthal anisotropy at asthenospheric depths^76^. Combining SKS splitting measurements and surface waveforms (including overtones) showed the presence of two layers of frozen-in anisotropy with an interface of varying depth in the lithosphere, and an increased azimuthal anisotropy at asthenospheric depths aligned with the APM^77^. Strong radial anisotropy with ξ > 1 is also found at asthenospheric depths in the western US^78^. Its complex pattern is attributed to the complex dynamics in this region^79–82^.

Figure 2 panel D compares the strength of the azimuthal anisotropy as a function of depth beneath the western and cratonic north American continent from the model of Yuan and Romanowicz^77^. The corresponding isotropic shear velocity (V_siso_) depth profile is also shown. dlnG represents the relative strength of the azimuthal anisotropy, referred to a reference isotropic model, where G is the amplitude of 2Ψ anisotropy as defined by Montagner and Nataf^83^ (Ψ is the azimuth). In the tectonically active western US (green lines) the lithosphere is thin, the LVZ shows a minimum in V_siso_ at ~ 100 km, and this minimum corresponds to the peak in the strength of azimuthal anisotropy. In contrast, beneath the craton (pink lines), the lithosphere is ~ 200–250 km thick, and the azimuthal anisotropy presents two peaks, one in the shallow lithosphere and another around 270 km depth in the LVZ. This pair of profiles illustrates the differences in anisotropy depth profiles under continents and suggests how olivine alignment in a young lithosphere may eventually be incorporated into the lithosphere of an older continent. Everywhere beneath the continent, the strength of anisotropy decreases to below resolution at depths larger than 300 km.

In summary, observations of both azimuthal and radial anisotropy at depths corresponding to the seismic LVZ, both under continents and oceans, suggest the present-day shearing in a weak asthenospheric channel extending from the base of the lithosphere to no more than 300 km depth (e.g. Fig. 2E).

## The asthenosphere, geodynamics, plate tectonics, and thermal-magmatic history

The asthenosphere has been considered an important component of plate tectonics since plates were first defined^84^. From the beginning it was thought that the asthenosphere might be a naturally evolving consequence and perhaps even a regulator of mantle convection, rather than an independent, planet specific, pre-condition for plate tectonics. The following paragraphs review studies that suggest that plate tectonics is promoted by a thinner asthenosphere, that either a non-linear rheology or the incorporation of recycled/subducted water might generate an asthenosphere almost automatically from mantle convection, and that water cycling could regulate mantle convection by controlling aspects of plate tectonics.

An indication that the dynamic role of the asthenosphere for mantle convection, and by association plate tectonics, comes from both its viscosity and thickness, came from geoid modeling and seismic studies that mapped long wavelength structure in mantle^3,85^ The existence of long wavelength mantle structure presented a dynamic problem as long wavelength structure is not expected at the degree of convective vigor of the Earth’s mantle. If asthenosphere viscosity is the only defining factor for its role in mantle dynamics, this only exasperates the problem as lower viscosity would mean greater convective vigor and a tendency toward shorter wavelength flow^86^. This conundrum motivated mantle convection simulations that explored a range of factors that could generate long wavelength mantle convection. One factor that was found to contribute within numerical models, albeit not necessarily the only factor that could, was the presence of an upper mantle layer with viscosity lower than plates above and bulk mantle below, i.e., a model analog for the asthenosphere^87,88^.

The simulations of Bunge et al.^87,88^, and subsequently others^89–92^, identified a connection between the asthenosphere and mantle dynamics but did not address how, physically, the asthenosphere could contribute to the existence of long wavelength flow in the Earth’s mantle. Theoretical scaling analysis showed that flow channelization within the asthenosphere is a critical factor^93,94^. This alters the vertical length scale of mantle shear, which affects the balance of lateral and vertical viscous dissipation in the mantle—viscous dissipation being a key limiter on flow wavelength. The analysis showed that the depth and the viscosity of the channelized region fed into flow wavelengths. More critically, for the theme of this paper, it showed that the presence of a relatively thin asthenosphere altered the energetic balance of the whole mantle. A subsequent analysis showed that this result is robust when dissipation associated with plate margins is included in the energy balance, i.e., the role of the asthenosphere in the global energetics of the solid Earth system is comparable to that of plates and plate margins^95^.

Although an asthenosphere can increase the wavelength of mantle convection it is not the only physical factor that can do so. In particular, geodynamic models have also shows that the strength of the lithosphere can also promote long-wavelength flow^96,97^. For the Earth, both factors are present and the combination can generate horizontal flow dimensions that are significantly greater than mantle depth^94,97^. Similarly, although flow channelization provides one physical interpretation of how long wavelength flow can be maintained, it is not the only analysis that provide physical insight into the dynamics of long-wavelength mantle convection. A channelization analysis cannot explain how long-wavelength flow is generated to begin with. The physics associated with that question has been elucidated via a Rayleigh–Taylor analysis^98^. That analysis showed how a strong lithosphere above a weak asthenosphere could favor the formation of long-wavelength instabilities and, by association, damp the growth of shorter wavelength instabilities. The key commonality to both a Rayleigh–Taylor and a channelization analysis is that they both indicate that an asthenosphere alters the global energetics of the mantle. The Rayleigh–Taylor analysis shows that it does so in a way that promotes the initiations of long-wavelength flow while the channelization analysis shows that it does so in a way that promotes the maintenance of long-wavelength flow.

**Figure 3:**
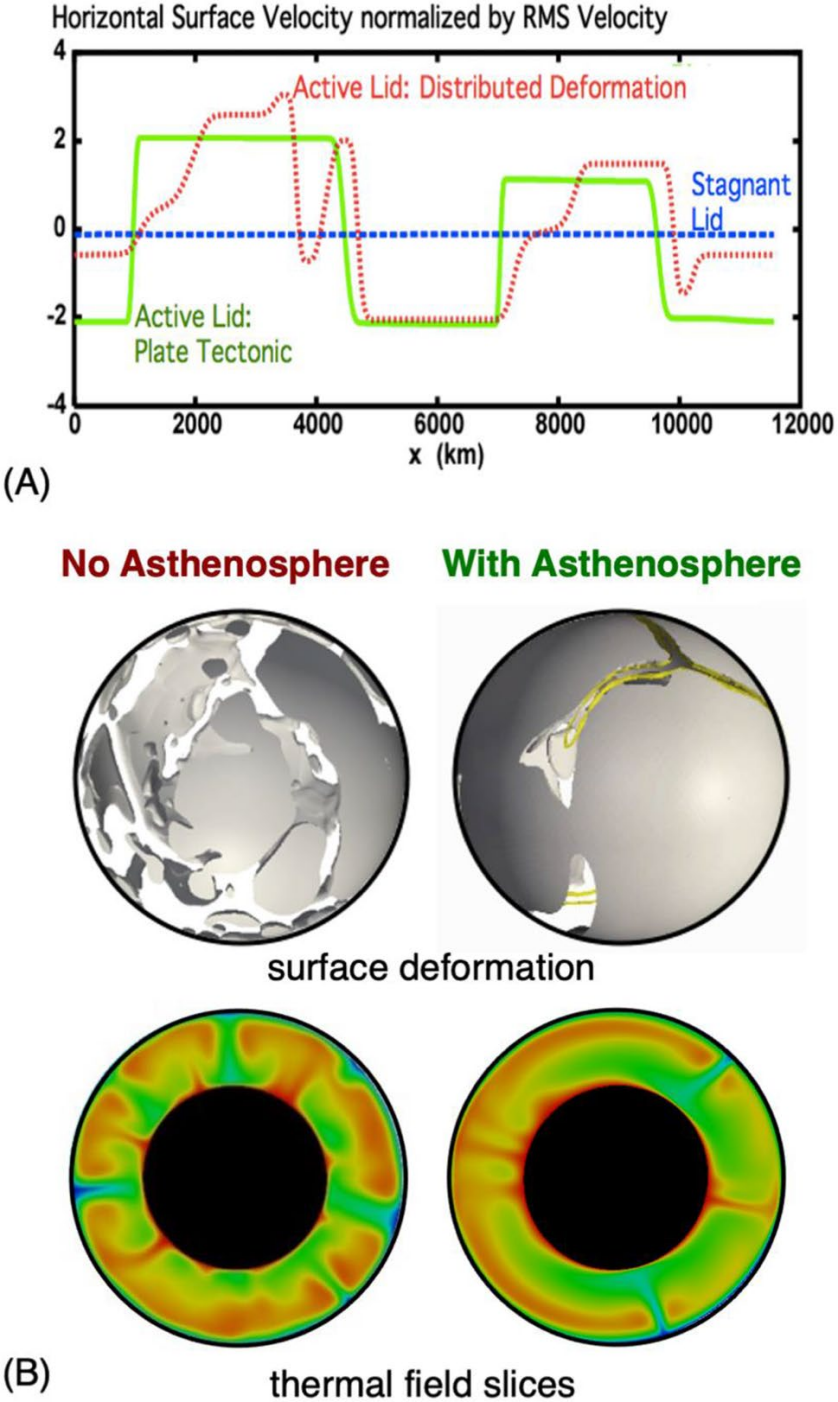
(**A**) Horizontal surface velocities from the models of Richards et al.^102^. (**B**) Results from the models of Hoink et al.^103^. Top images show surface deformation patterns. Solid grey regions represent regions that move coherently with no internal deformation (model analogs to tectonic plates). White/transparent zones are regions of distributed lithosphere deformation. Yellow lines represent regions of concentrated lithosphere deformation (deformation zone with extents comparable to lithosphere thickness). Lower images show thermal slices through the models (blue is cold, red is hot).

The rheology of plates and the energetics of plate deformation have received significant attention in efforts to extend plate tectonics from a kinematic to a dynamic theory^99,100^. Certainly, if weak plate margin zones could not form, then lithosphere strength would prevent it from being able to subduct and the Earth would be a single plate planet^101^. However, the existence of lithospheric weak zones does not necessarily lead to a plate tectonic mode of mantle convection. A weak lithosphere can participate in mantle overturn, referred to as an active lid mode of mantle convection, but plate tectonics is more than lithosphere overturn. Plate tectonics requires the presence of internally rigid plates separated by narrow zones of deformation. Richards et al.^102^ first showed, using 2-D Cartesian simulations, that the presence of an asthenosphere facilitated a plate tectonic mode of convection. Subsequently, Hoink et al.^103^ showed that this result was robust in 3-D spherical geometry. Figure 3 shows results from those two studies.

Figure 3a shows horizontal surface velocities from three representative cases of Richards et al.^102^. If the lithosphere is so strong that it cannot be deformed, then mantle convection occurs below a coherent lithosphere that does not participate in mantle overturn. This is a model analog of a single plate planet, also referred to as a stagnant-lid mode of mantle convection, with a uniform surface velocity near zero. If the lithosphere can deform, then regions of weakness can develop that allow the lithosphere to overturn. However, in cases without an asthenosphere, Richards et al.^102^ found that the prevalent mode of convection was one with broad zones of distributed deformation and surface velocities not akin to present day plate tectonics. Introducing an asthenosphere led to piecewise constant surface velocities, the essential feature of plate tectonics.

Figure 3b shows representative cases from Hoink et al.^103^. The 3-D spherical models confirmed the general conclusion that an asthenosphere layer contributed to a plate tectonic mode of mantle convection. The study also showed that a relatively thin asthenosphere (~ 100–200 km) allowed a plate tectonic mode to exist over a wider range of lithosphere strength than cases with a thicker low viscosity layer in the mantle. As with long wavelength mantle structure, flow channelization below the lithosphere played a key role for maintaining a plate tectonic mode of convection. Stated another way, the asthenosphere can be dynamically defined as a region of channelized upper mantle flow. That a thinner region of channelization favors a plate tectonic mode of mantle convection suggests that the lithosphere/asthenosphere boundary (LAB) is, in effect, the largest “plate boundary” on Earth.

Defining the asthenosphere as a region of flow channelization provides a direct connection to seismic observations (Seismic Anisotropy Section). Mineral physics indicates that upper mantle viscosity is non-Newtonian (a power law viscosity that displays shear weakening) while the lower mantle is Newtonian^104,105^. A non-Newtonian upper mantle allows low viscosity regions to emerge in response to mantle flow^106,107^. King^108^ showed how a non-Newtonian upper mantle could lead to depth-variable effective mantle viscosity, consistent with post glacial rebound and geoid constraints, through the dynamic formation of a low viscosity layer in the upper mantle. Semple and Lenardic^109^ used a similar approach to connect the asthenosphere, as an emergent zone of channelized flow, to seismic observations and further to suggest that asthenosphere flow could be a driver of plate motion.

The study of Semple and Lenardic^109^ was motivated by the seismic experiment of Lin et al.^58^, subsequently confirmed by Russell et al.^59^. Lin et al.^58^ detected two distinct, localized regions of seismic anisotropy below an oceanic plate. One was at the base of the lithosphere. The other was roughly 200 km below that and was taken to indicate the base of the asthenosphere. Lin et al.^58^ also noted that their results were consistent with the idea that pressure-driven asthenosphere flow can act as a plate-driving force, as previously argued for by Hoink et al.^110^. Hoink et al.^110^ determined the conditions under which the asthenosphere could transition from providing low resistance to plate motion to providing a plate-driving force. Semple and Lenardic^109^ showed that those general predictions held up for models with non-Newtonian upper mantle viscosity, and that model predictions, based on flow channelization and its effects on olivine alignment, were consistent with the seismic results of Lin et al.^58^. More specifically, pressure-driven flow, in a non-Newtonian region of channelization, could lead to a plug-flow configuration with concentrated zones of shear deformation at its top and at its base. The shear zones would lead to alignment of olivine that would contribute to seismic anisotropy. The pressure driven plug-flow would also locally act as a plate-driving force—distinctly different from the canonical idea that the role of the asthenosphere for plate tectonics comes only from its low resistance to plate motions.

The idea that channelized asthenosphere flow could locally act as a plate driving force did not initially gain traction as observational support was relatively slim at the time it was proposed^110^. That has progressively changed^58,59,111–116^. The accumulation of observations that question the canonical view of the asthenosphere’s role for plate tectonics have fed into the development of more refined numerical simulations that connect mantle convection to plate tectonics, with the asthenosphere playing a key role^117–119^.

Our discussion thus far has not addressed the role of the asthenosphere for the Earth’s geologic history spanning billions of years. Tectonics, geology, and magmatism/volcanism are all ultimately connected to the Earth’s internal energy, and their evolution depends on the Earth’s thermal history, i.e., how the Earth’s interior has cooled over geologic time. Surprisingly, from our perspective, the majority of thermal history models to date have not made explicit connections to the asthenosphere. This provides opportunity for future work. As a motivation for that, we will end this section with a review of a recent thermal history study that was motivated by a long-standing observation connected to the asthenosphere.

**Figure 4:**
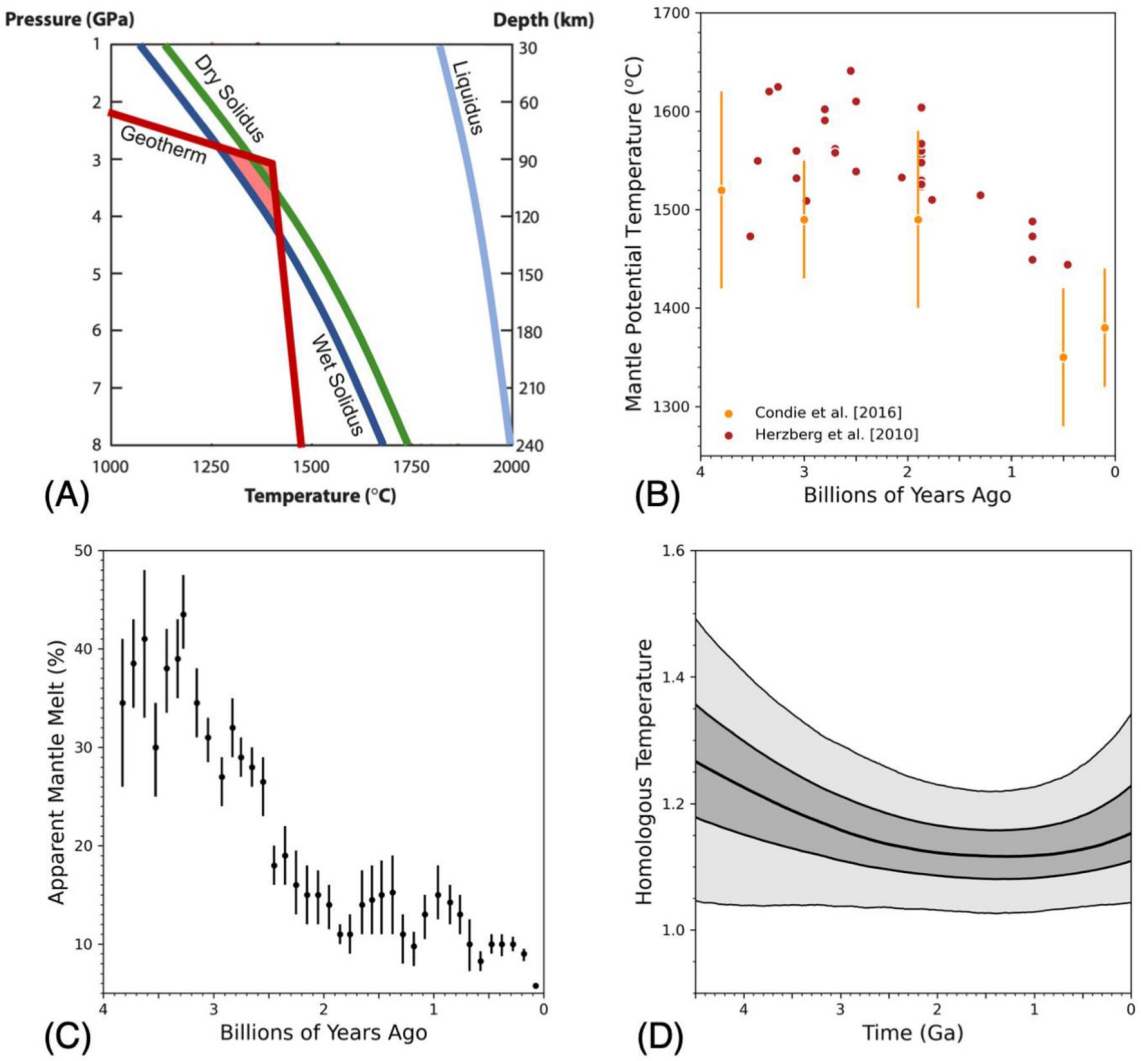
(**A**) Mean oceanic geotherm (red) relative to the dry (green) and wet solidus (dark blue) and liquidus (light blue) of the upper mantle. The geotherm is based on Stein and Stein^134^. The dry solidus is from Hirschmann^135^. The liquidus and wet solidus, assuming 2.5 ocean masses in the mantle, are from Katz et al.^121^). (**B**) Petrologic data constraints on mantle potential temperature versus time^124,125^. (**C**) Apparent mantle melt fraction from continental arcs over geologic history^126,127^. (**D**) Results from coupled thermal and deep-water cycling evolution models for homologous asthenosphere temperature over geologic time^123^. The darker color envelope highlights values falling between the upper and lower quartiles from the statistical distribution of all model cases that can match thermal data, within data and model uncertainties. The lighter color envelope constrains the maximal and minimal limits.

It has long been noted that the temperature of Earth's asthenosphere is remarkably close to the melting temperature of rock, as plotted in Fig. 4a ^120^. That proximity is critical to Earth's magmatic activity and to the asthenosphere being the dominant source of igneous rocks on our planet at present. It could also connect the asthenosphere to the Earth’s magmatic history not only through temperature (thermal history) but also through water content, which has a significant effect on mantle melting temperature^121^. The dependence of mantle viscosity on hydration provides an added connection as it is another factor that could contribute to the formation and maintenance of an asthenosphere^122^. The connections, and feedbacks between mantle melting, mantle hydration and Earth cooling, have recently been explored in coupled thermal history and deep-water cycling models^123^.

Figure 4b shows petrological constraints on mantle cooling that any thermal history model must match. There is spread and uncertainty in the data but a consistent trend is that mantle cooling is mild from 4 to 2 Ga and then accelerates from 2 Ga to the present day^124,125^. Figure 4c shows petrological constraints on mantle melting over geologic time^126,127^. Again, there is uncertainty but, as with the cooling data trend, there is a break in behavior at ~ 2 Ga. What is striking is that over a time window during which mantle temperatures remained nearly constant, mantle melt percentage declined significantly and over a time window when mantle temperature dropped steeply, melting behavior remained nearly constant. This is difficult to explain if the evolution of melting in the asthenosphere is due solely to changing thermal conditions. Seales et al.^123^ hypothesized that the trends could be self-consistently explained by the co-evolution of mantle cooling and deep-water cycling.

Seales et al.^123^ developed a methodology to directly incorporate thermal history constrains into previous deep water cycling models^128–131^. This allowed 10’s of thousands of data-constrained models to be calculated. A robust model trend is plotted in Fig. 4d. The models tracked a homologous temperature defined as the ratio of sub-lithosphere temperature to melting temperature within the asthenosphere, both of which evolved over model time with the later dependent on the water content evolution of the asthenosphere. The initial drop in homologous temperature, in the face of near constant mantle temperature, was due to the preferential trend of mantle dewatering at higher temperatures. Dewatering also increases mantle viscosity which lowers mantle heat loss and helped to maintain a constant temperature. That trend could not be maintained as the mantle could not continue to dehydrate beyond a limit set by initial water content. That in turn initiated a cooling trend. Under cooler temperatures, recycling of water into the mantle becomes more efficient^128,132^. This initiated the phase of near constant homologous temperature plotted in Fig. 4d.

Figure 4d shows that the ratio of asthenosphere temperature to melting temperature can be buffered over a billion-year time scale. That is, feedbacks between mantle cooling, deep-water cycling and the effects of temperature and water content on asthenosphere viscosity can cause the geotherm and the solidus to co-evolve and become pegged to each other. This can maintain sub-lithospheric partial melting and a melt-rich asthenosphere^133^. To state this from a somewhat different perspective, the overall implication is that the persistence of plate tectonics, as facilitated by the existence of the asthenosphere, is in no small part due to the self-regulating effect of deep mantle water cycling. Furthermore, to the degree that asthenosphere properties depend on melting and water content (acknowledging that other factors may also play a role), the asthenosphere can be self-regulating in a manner that has first-order effects on the Earth’s thermal and magmatic history. The models reviewed assumed that plate tectonics was operative over the Earths geologic history but extension of the work can address the question of how asthenosphere self-regulation connects to the onset time of plate tectonics and how it affects the subsequent maintenance of plate tectonics.

## Conclusions

Many lines of evidence indicate a thin, weak asthenosphere that is ubiquitous under continents and oceans, and that fundamentally influences Earth dynamics. Small dimension (short-wavelength) loads attain isostatic equilibrium far faster than might otherwise be suggested by large-scale glacial isostatic adjustment. This requires an asthenosphere beneath mature continental lithosphere. Post-seismic rebound provides a direct probe of asthenosphere viscosity near plate boundaries. Shearing in the asthenosphere, by plate motions, aligns olivine crystals and produces seismic anisotropy that is aligned with the absolute motion of oceanic plates. Pressure-driven flow in the asthenosphere can also lead to alignment of olivine at the top and bottom of the asthenosphere and the direction of alignment can be different from the absolute plate motion. Seismic anisotropy indicates that an asthenosphere underlies both continents and oceans. Plate tectonics as we know it on Earth is facilitated by an asthenosphere, and is especially promoted by a very thin and weak asthenosphere. An asthenosphere can develop as a natural consequence of mantle convection in which water is recycled into the Earth’s interior by overturning lithosphere, and with pervasive sub-lithospheric partial melting. A non-Newtonian upper mantle rheology, in addition to water and melt, can enhance the dynamic development of an asthenosphere, which in turn maintains plate tectonics and affects planetary heat loss. The evidence for an asthenosphere and its influence on earth dynamics, as reviewed in this paper, seems to us remarkably consistent, compelling, and interesting. We see no firmly contradictory evidence, and lots of suggestions for future work. Particularly interesting, in terms of asthenosphere structure, would be to determine whether the thickness and viscosity of the asthenosphere are similar everywhere. In terms of asthenosphere dynamics, much work remains to understand its full influence on the history and evolution of plate motions, continental aggregation and dispersal, the dynamics of plate boundaries, and the geochemical/ petrological evolution of the mantle.

### Supplementary Information

Supplementary Information.

## Data Availability

All data used is provided in the Supplemental Material.
