# Supplementary material for: Influence of the asthenosphere on earth dynamics and evolution
Source: Sci Rep. 2023 Aug 17;13:13367. doi: 10.1038/s41598-023-39973-y (PMC10435468; doi:10.1038/s41598-023-39973-y)
Supplement: Supplementary file 1 — Supplementary Information. [file 41598_2023_39973_MOESM1_ESM.docx]

# Supplemental Material

## Supplemental Material S1

The response of a viscous earth overlain by a lithosphere to loading is:

$h\left(t\right)=h_{0}\alpha^{-1}e^{\frac{-t}{\tau}}$ (1)

where $\tau=\tau_{m}\alpha^{-1}R, \tau_{m}=2\eta_{m}k/\rho g, k=2\pi/\lambda=\frac{n+0.5}{r_{earth}}, \alpha=1+\frac{k^{4}F_{r}}{\rho g}$, λ is the load wavelength, k is the wave number, n is the order number, ρ(kg m^-3^)=3.313 is the adiabatic density of the upper and lower mantle, and g(m s^-1^)=9.832. *τ_m_* is the response time of the mantle with no lithosphere or asthenosphere, *F_r_* is the flexural rigidity of the lithosphere, and *R* is the ratio of the response time of the asthenosphere to *τ_m_*. *R* is an analytic function of *k,* the thickness of the asthenosphere (or upper mantle)*, H_asth_,* and the ratio of the asthenosphere viscosity to the viscosity of the underlying mantle half space, *η_asth_/η_m_* (from Cathles [1], equation III-21).

The PREM flexural rigidity is computed from the elastic Lame parameters and thickness of the crust and lid in PREM. $F_{r}^{PREM}=53.2\times10^{23}Nm$. The thickness of the PREM lithosphere (not counting the 3 km ocean layer) is $T_{ePREM}=77 km$, and $T_{e}/T_{ePREM}=\left(F_{r}/F_{r}^{PREM}\right)^{1/3}$. The model computes the response to loading in terms of the load wave number k. The wave number *k_c_* that characterizes the response of a load of dimensions L by W is (from *Cathles* [1], App VI)

$k_{c}=1.7\left(L^{-2}+W^{-2}\right)^{0.5}$. (2)

Equation (1) is accurate for order numbers greater than ~11 (Cathles [1], Fig. IV-26). At order numbers smaller than ~11, motions of the core-mantle boundary and changes in gravity would be important, but since we are concerned in this paper with the asthenosphere and upper mantle, this low order number response is not important to the discussion in this paper.

## Supplemental material S2

McConnell [2] Hankel transformed emerged shorelines mapped by Sauramo [3] to determine the exponential response time as a function of wavelength. McConnell’s spectrum shows the rapid uplift in the central Gulf of Bothnia area noted by Sauramo as a small peak with a decay time of ~1 ka and a wave number of 1.2x10^-2^ km (lower panel Figure S2.1), which by equation S1 (2) suggests a load of radius 100 km.


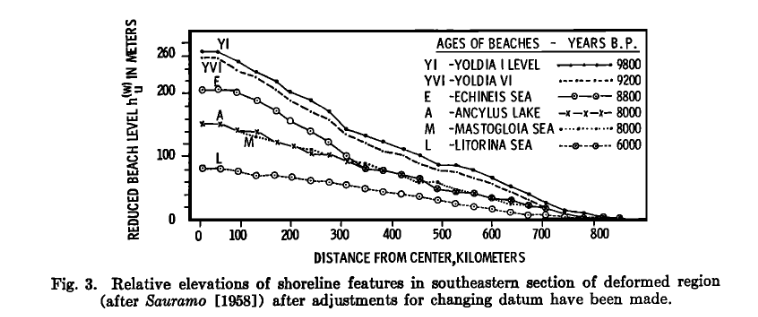


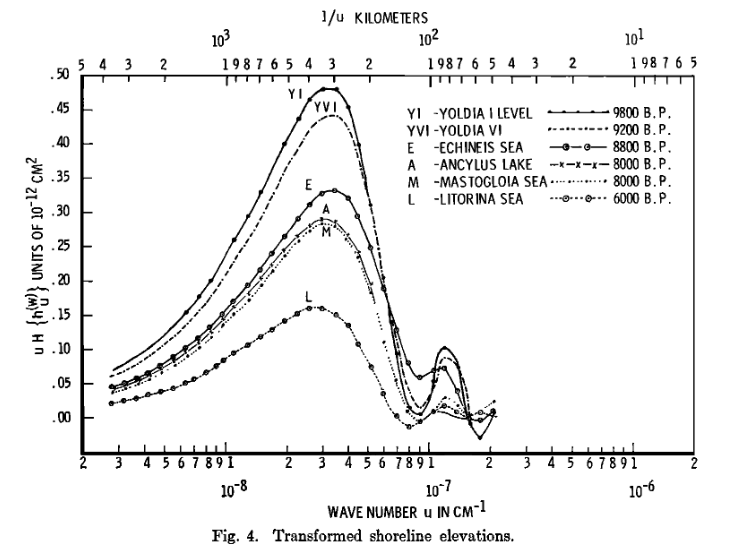


*Figure S1. Figures from McConnell [2]. (top) Radially symmetric average of Sauramo’s emergence data. Note very rapid initial uplift of central area. (bottom) Hankel transform of the shorelines, H, multiplied by wave number k (note McConnell’s wave number symbol is u). The rapidly responding central portion is the small second peak.*

The inverse transform of the curves in S2.1 (bottom panel) is: $e(r,t_{n})=\int_{k=0}^{\infty} H(k,t_{n})J_{0}(kr)k dk$, where e(r,t_n_) are the emergence profiles in S2.1 (top panel). If we are interested in only the central location, r=0 and $J_{0}(0)=1$, so we can numerically integrate McConnell’s curves and distinguish the emergence related to the large peak from that related to the smaller peak, with the results shown in Table S2.1. The small peak contributes about 37m of emergence to the total central emergence of 251m.

*Table S2.1 Inverse transform of Figure I.1 (bottom) at r=0*

|  | YV1 |  | E |  | M |  | L |
| --- | --- | --- | --- | --- | --- | --- | --- |
| age[ka] | 9.2 |  | 8.8 |  | 8.0 |  | 6.0 |
| Integrl | 250.8 |  | 208.5 |  | 138.9 |  | 74.0 |
| main pk | 213.9 |  | 182.5 |  | 129.3 |  | 67.5 |
| small pk | 36.9 |  | 34.1 |  | 9.5 |  | 6.5 |


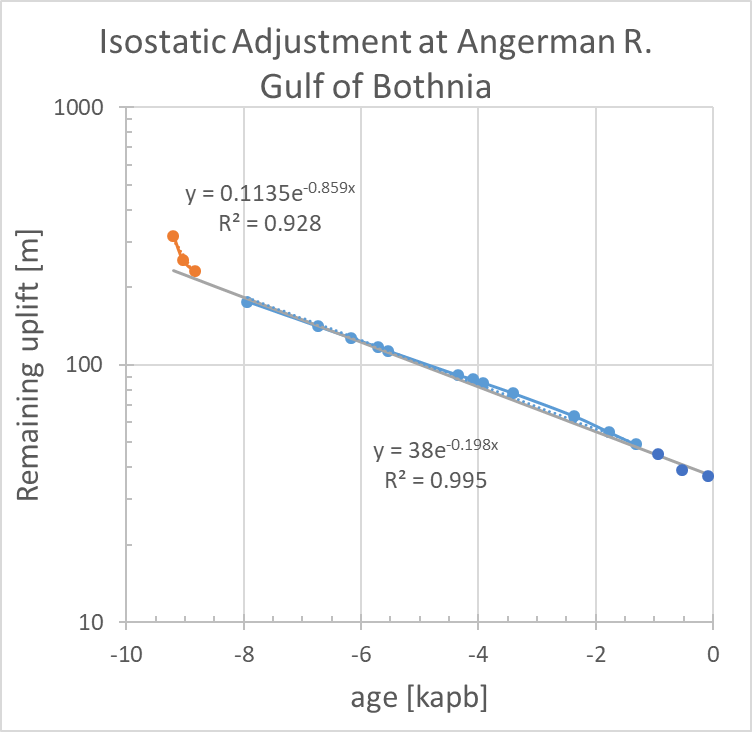


*Figure S2. Remaining uplift at the mouth of the Angerman River in the Gulf of Bothnia from Liboutry [4]. Gray line is determined is fit to the data points connected by the blue line*

The independently-determined central uplift at the Angerman River in the Gulf of Bothnia, plotted in Figure S2.2, shows the same rapid initial uplift (red data points), with nearly the same decay time, (1.16 ka) as determined by McConnell. The longer wavelength central response has a decay time of 5 ka, and there is 38 m of uplift remaining.

Assuming T_ePREM_=26, τ=1ka, and L=W=200 km (roughly the dimensions of the Gulf of Bothnia area where the uplift is anomalously rapid), the viscosity of the half space needed to match the response time is 4.8x10^19^ Pa s, and the required viscosity of a 75 km thick asthenosphere overlying a 10^21^ Pa s mantle is 1x10^19^ Pa s.

## Supplemental Material S3

The C_t_ parameter developed in Richards and Lenardic [5] shows the equivalency of a great many “asthenospheres” by determining how the asthenosphere thickness trades off against asthenosphere viscosity in the limit where the interrogating wavelength is large with respect to the asthenosphere thickness. Equation S1(1) can be used in the scaling spirit of C_t_ to examining the equivalency of published GIA models. It has no wavelength constraint and includes the impact of the lithosphere on isostatic adjustment. The spectral response of 23 Fennoscandian earth models compile by Steffen and Wu [6] are tabulated in Table S3.1 and plotted in Figure S3.1.

*Table S3.1. Summary of 23 GIA models from Steffen and Wu [6] with F_r_ and the viscosity ratio added. The upper mantle is assumed to be 650 km thick.*

| **Reference** | **T_e_[km]** | **F_r_[10^23^ Nm]** | **η_UM_[10^21^ Pa s]** | **η_LM_[10^21^ Pa s]** | **η_UM_/η_LM_** |
| --- | --- | --- | --- | --- | --- |
| Lambeck et al. (1998b) | 75 | 49 | 0.3 | 5 | 0.060 |
| Kaufmann and Lambeck (2002) | 120 | 202 | 0.7 | 20 | 0.035 |
| Steffen and Kaufmann (2005) | 120 | 202 | 0.4 | 100 | 0.004 |
| Steffen and Kaufmann (2005) central | 160 | 478 | 0.4 | 100 | 0.004 |
| Steffen and Kaufmann (2005) peripheral | 100 | 117 | 0.4 | 70 | 0.006 |
| Lambeck et al. (1998a)#1 | 100 | 117 | 0.4 | 5 | 0.080 |
| Lambeck et al. (1998a)#2 | 80 | 60 | 0.45 | 5 | 0.090 |
| Wieczerkowski et al. (1999) | 95 | 100 | 0.48 | 5.9 | 0.081 |
| Milne et al. (2001) | 120 | 202 | 0.65 | 80 | 0.008 |
| Fleming et al. (2003) | 110 | 155 | 0.5 | 7.2 | 0.069 |
| Klemann and Wolf (2005) | 80 | 60 | 0.5 | 2.4 | 0.208 |
| Lambeck et al. (1998a)#3 | 70 | 40 | 0.3 | 20 | 0.015 |
| Lambeck et al. (1998a)#4 | 80 | 60 | 0.35 | 30 | 0.012 |
| Lambeck et al. (1998a)#5 | 110 | 155 | 0.5 | 20 | 0.025 |
| Davis et al. (1999)#1 | 156 | 443 | 0.72 | 20 | 0.036 |
| Davis et al. (1999)#2 | 153 | 418 |  | 20 |  |
| Milne et al. (2001,2004) | 120 | 202 | 0.8 | 10 | 0.080 |
| Milne et al. (2004) | 120 | 202 | 1 | 2 | 0.500 |
| Bergstrand et al. (2005) | 120 | 202 | 0.5 | 30 | 0.017 |
| Steffen and Kaufmann (2005)#2 | 120 | 202 | 0.7 | 10 | 0.070 |
| Lidberg et al. (2010) | 120 | 202 | 0.5 | 5 | 0.100 |
| Steffen et al. (2010)#1 | 160 | 478 | 0.4 | 10 | 0.040 |
| Steffen et al. (2010)#2 | 160 | 478 | 0.4 | 20 | 0.020 |


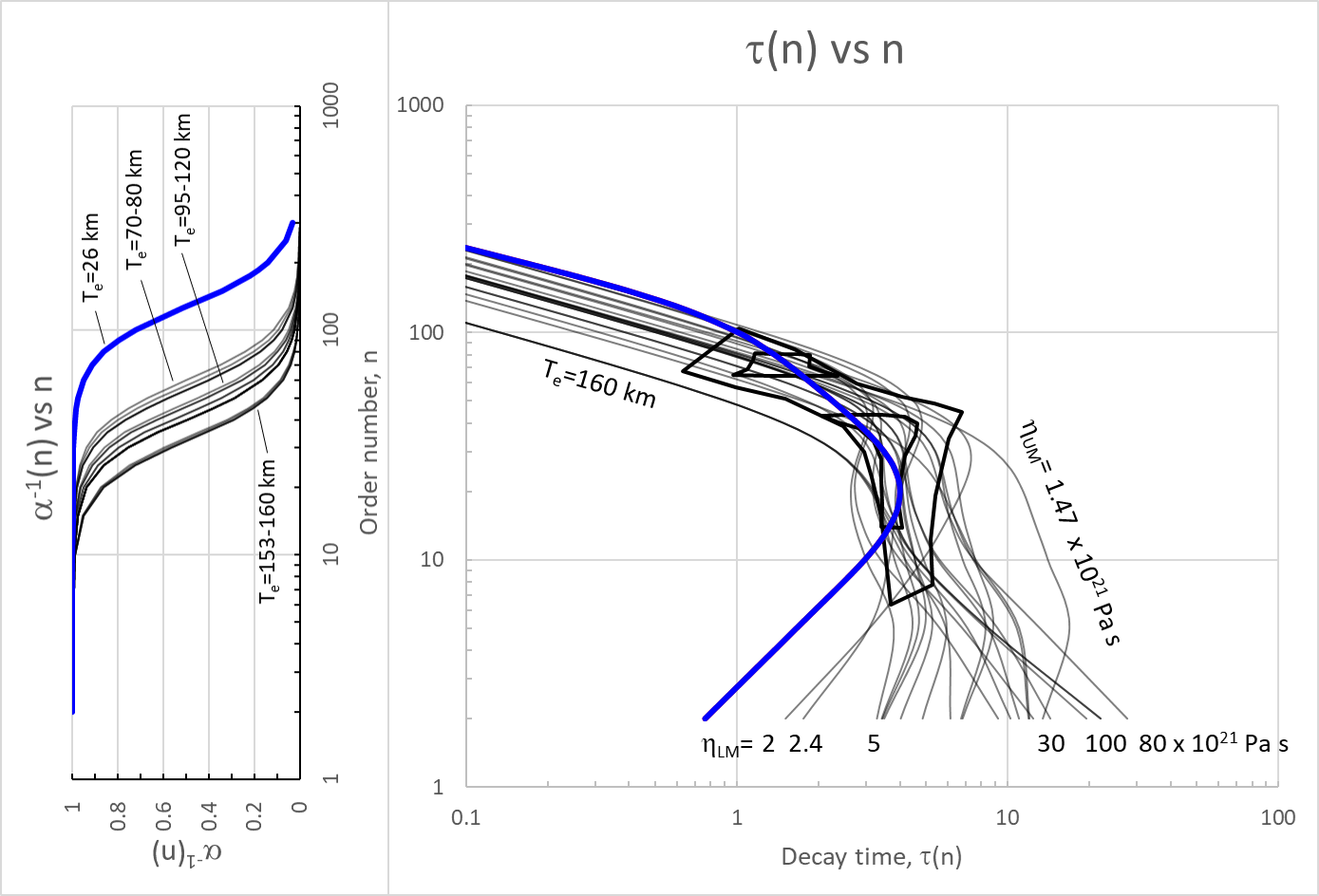


*Figure S3. Grey curves computed using S1(1) from parameters in Table S3.1. (left panel) load filter. (central panel) decay spectra. Blue curve is “F” earth model from text Figure 1. The upper mantle layer is 650 km thick in all grey-curve cases. Their lower mantle viscosity and lithosphere flexural rigidity are as listed in Table S3.1. The blue curve asthenosphere is 1.5x10^20^ Pa s and 75 km thick, its underlying mantle is 1x10^21^ Pa s.*

## Supplemental Material S4

*Table S4.1. Decay times τ and corresponding order numbers for locations shown in Figure 1b. Load dimensions W (=width of square-edged bar long in other direction) and R_o_ (the radius of cylindrical load) determine the effective wave number according to k_c_=1.2/R_o_ or 1.7/W. The order number is determined from the relationship k=2π/λ=(n+0.5)/r_earth_. Subscript SP means calculated from spectral peak. Subscript M means calculated from earth model using equation S1 (1). Subscript 50% means T_e_ is estimated assuming less than 50% of the surface load is supported by the lithosphere. Methods discussed in Cathles [1], Appendix VI).*

| **Area** | **Label** | **m emerg.** | **τ[ka)** | **Size (km)** | **T_e_(km)** | **n** | **Reference** (or p. Cathles [1]) |
| --- | --- | --- | --- | --- | --- | --- | --- |
| **S. Shetland Islands** | Ant | ~7 | 0.07 | W=60 | 15 | 180 | Simms et al. [7] |
| **Meisters Vig, Gnld.** | G_MV_ | 80 | 1 | W=60 | 16_50%_ | 180 | Cathles [1], p. 164 |
| **SE Alaska** | Alk | ~5 | 0.75_M_ | R_o_=139_SP_ | 50 | 54.3_SP_ | Larsen et al. [8] |
| **Devon Isl., Canada** | D | 85 | 1 | W=60 | 16_50%_ | 180 | Muller and Barr [9], Margold et al. [10] |
| **Lake Bonneville** | B | 65 | 0.83 | R_o_=100 | 20 | 76 | Passey [11] |

## Supplemental Material S5

Table S5.1 summarizes the recent ice loading histories, observed uplift rates, and inferred asthenosphere viscosities in selected well-studied localities. If isostatic adjustment is very fast, the uplift tracks the deglaciation rate indicated in the column headed dh_L_/dt. The viscosity of the asthenosphere slows the response to the observed uplift rate indicated in the column labeled r_obsv_. Typically the authors compute many earth models to determine the asthenosphere viscosity indicated in the η_asth_ column. We calculate the no-lithosphere decay time τ(ka) from this asthenosphere viscosity and the viscosity of the underlying mantle indicated by the authors. Asthenosphere viscosity decreases slightly as lithosphere thickness increases (Auriac et al, [12]), and the decay time is greatest when no lithosphere is present. Therefore in text Figure 1, we plot response time for the maximum asthenosphere viscosity cited by the authors at the order number appropriate to the dimensions of the unloaded area, and attach an arrow pointing to the shorter response times that a lithosphere would induce.

*Table S5.1. Determination of no-lithosphere decay time that produces the observed present-day uplift rates at selected localities of recent or post LIA deglaciation. Load(t) arrays show the deglaciation history where the first column is time in (ka) and the second column is the load at that time expressed in m of mantle (ρ=3313 kg m^-3^). dh_L_/dt is the most recent rate of load change (deglaciation rate) in mm/y, and the next column is the observed rate of uplift. The dimensions of the unloaded area are given in the next column. The order number n is computed from the load dimensions as explained in S1. η_asth_ is the largest best inversion viscosity of the authors (typically that with the weakest lithosphere). τ_no lith_(ka) is the response time calculated for η_asth_ with no lithosphere. T_e_(km) is the effective thickness of the lithosphere relative to PREM (converted from the effective thickness cited by the authors to be compatible with the elastic parameters of PREM, if necessary).*

| Locality | Load(t) | dh_L_/dt(mm/y) | r_obsv_(mm/y) | LxW (km) | n | η_asth_(10^21^ Pa s) | τ_no lith_(ka) | T_e_(km) | reference |
| --- | --- | --- | --- | --- | --- | --- | --- | --- | --- |
| Patagonia A | ¯0.143 0  ¯0.07 ¯19.7 ¯0.038 ¯23.2 ¯0.019 ¯27.2  0 ¯32.1 | 260 | 41 | 50x250 | 220 | 0.0016 | 0.108 | 39.5 | Richter et al. [13] |
| Patagonia B | ¯0.383 0  ¯0.143 ¯0.05 ¯0.07 ¯12  ¯0.038 ¯15.7  ¯0.019 ¯20.5  0 ¯32.4 | 623 | 41 | 50x250 | 220 | 0.008 | 0.54 | 39.5 | Richter et al. [13] |
| Iceland | ¯0.12 0  0 ¯11 | 93 | 23 | 120x120 | 127 | 0.004 | 0.39 | 13-35 | Auriac et al. [12] |
| S. Shetland Islands | ¯0.72 0 ¯0.43 19 ¯0.23 0  0 0 | 96 | 6m†  <7.5 mm/y | 62x700 | 174 | 0.002 | 0.11 | 14-32 | Simms et al. [7] |
| Amundsen Sea | ¯0.102 0  ¯0.012 ¯10.7  0 ¯15.7 | 429 | 41 | 125x750 | 87 | 0.016 | 0.14 | 60 | Barletta et al. [14] |
| N. Norway | ¯5.3 0 ¯0.3 5  0 0 | 17 | 2.5 | 150x700 | 73 | 0.018 | 2 | 26 | Fjeldskaar (unpub) |

† The constraints for this case are that the post LIA uplift be ~6 m and the present rate of uplift<7.5 mm/y. Both are important constraints but the shoreline emergence seems the most persuasive, so this data point is plotted as a blue box in text Figure 1.

## References

[1] Cathles, Lawrence M. (1975). *The Viscosity of the Earth’s Mantle*. Princeton: Princeton University Press.

[2] McConnell, R.K., (1968). Viscosity of the mantle from relaxation time spectra of isostatic adjustment. J. Geophys. Res. 73, 7089–7105. https://doi.org/10.1029/JB073i022p07089

[3] Sauramo M. (1958). Die Geschichte der Ostee. *Annales Academae Scientiarum Fennicae* *Ser. A III*, 51.

[4] Lliboutry, L.A., (1971). Rheological properties of the asthenosphere from Fennoscandian data. J. Geophys. Res. 76, 1433–1446. https://doi.org/10.1029/JB076i005p01433

[5] Richards, M. A., A. Lenardic, (2018). The Cathles Parameter ( Ct ): A Geodynamic Definition of the Asthenosphere and Implications for the Nature of Plate Tectonics. Geochemistry, Geophysics, Geosystems. https:/doi.org/10.1029/2018GC007664

[6] Steffen, H., P. Wu, (2011). Glacial isostatic adjustment in Fennoscandia—A review of data and modeling. Journal of Geodynamics 52, 169–204.

[7] Simms, Alexander R., Erik R. Ivins, Regina DeWitt, Peter Kouremenos, and Lauren M. Simkins, (2012). Timing of the Most Recent Neoglacial Advance and Retreat in the South Shetland Islands, Antarctic Peninsula: Insights from Raised Beaches and Holocene Uplift Rates. *Quaternary Science Reviews,* *47 (July)*, 41–55. <https://doi.org/10.1016/j.quascirev.2012.05.013>.

[8] Larsen, Christopher F., Roman J. Motyka, Jeffrey T. Freymueller, Keith A. Echelmeyer, and Erik R. Ivins, (2004). Rapid Uplift of Southern Alaska Caused by Recent Ice Loss: Rapid Uplift of Southern Alaska. *Geophysical Journal International* 158 (3): 1118–33. <https://doi.org/10.1111/j.1365-246X.2004.02356.x>

[9] Muller, F., and W. Barr, (1966). Postglacial Isostatic Movement in Northeastern Devon Island, Canadian Arctic Archipelago. *ARCTIC* 19 (3): 263–169. https://doi.org/10.14430/arctic3433.

[10] Margold, Martin, Chris R. Stokes, and Chris D. Clark, (2018). Reconciling Records of Ice Streaming and Ice Margin Retreat to Produce a Palaeogeographic Reconstruction of the Deglaciation of the Laurentide Ice Sheet. *Quaternary Science Reviews* 189 (June): 1–30. <https://doi.org/10.1016/j.quascirev.2018.03.013>

[11] Passy, Q.R., (1981). Upper mantle viscosity derived from the difference in rebound of the Provo and Bonneville shorlines: Lake Bonneville Basin, Utah. *Journal of Geophysical Research, 86(B12)*, 11701-11708.

[12] Auriac, A., K. H. Spaans, F. Sigmundsson, A. Hooper, P. Schmidt, and B. Lund, (2013). Iceland Rising: Solid Earth Response to Ice Retreat Inferred from Satellite Radar Interferometry and Visocelastic Modeling. *Journal of Geophysical Research: Solid Earth,* *118 (4)*, 1331–44. <https://doi.org/10.1002/jgrb.50082>.

[13] Richter, A., E. Ivins, H. Lange, L. Mendoza, L. Schröder, J.L. Hormaechea, G. Casassa, et al., (2016). Crustal Deformation across the Southern Patagonian Icefield Observed by GNSS. *Earth and Planetary Science Letters* 452 (October): 206–15.

[14] Barletta, Valentina R., Michael Bevis, Benjamin E. Smith, Terry Wilson, Abel Brown, Andrea Bordoni, Michael Willis, et al., (2018). Observed Rapid Bedrock Uplift in Amundsen Sea Embayment Promotes Ice-Sheet Stability. *Science,* *360 (6395)*, 1335–39. <https://doi.org/10.1126/science.aao1447>
